# Development of novel reduced graphene oxide/metalloporphyrin nanocomposite with photocatalytic and antimicrobial activity for potential wastewater treatment and medical applications

**DOI:** 10.1038/s41598-024-77734-7

**Published:** 2024-11-13

**Authors:** Ahmed M. El-Khawaga, Hesham Tantawy, Mohamed A. Elsayed, Ahmed I. A. Abd El-Mageed

**Affiliations:** 1 Department of Basic Medical Sciences, Faculty of Medicine, Galala University, Galala City, 43511 Suez Egypt; 2 Head of Chemical Engineering Department, Military Technical College (MTC), Egyptian Armed Forces, Cairo, Egypt; 3 Chemical Engineering Department, Military Technical College (MTC), Egyptian Armed Forces, Cairo, Egypt; 4 Chemistry Department, Faculty of Science, Galala University, Galala City, 43511 Suez Egypt; 5 Colloids and Advanced Materials Group, Chemistry Department, Faculty of Science, Minia University, Minia, 61519 Egypt

**Keywords:** Reduced graphene oxide, Metalloporphyrins, Antimicrobial activity, Photocatalysis, Nanocomposite, Water treatment, Nickel, Methyl orange, Catalysis, Chemical biology, Environmental chemistry, Photochemistry, Surface chemistry

## Abstract

This research investigates a novel nanocomposite material composed of reduced graphene oxide (rGO) and nickel-5,15-bisdodecylporphyrin (Ni-BDP) nanoparticles for the effective removal of methyl orange (MO), a harmful synthetic dye, from water. The structure and composition of the synthesized rGO/Ni-BDP nanocomposite were characterized using high-resolution transmission electron microscopy (HR-TEM), Raman spectroscopy, nuclear magnetic resonance (NMR) spectroscopy, mass spectrometry, and ultraviolet-visible (UV-Vis) spectroscopy. The study demonstrates the material’s efficacy as both a catalyst and adsorbent for MO removal. Optimal performance was observed at pH 3.0, where the positively charged nanocomposite surface facilitated strong interactions with negatively charged MO molecules, leading to enhanced photocatalytic activity. Under these conditions, 0.01 g of the nanocomposite achieved an impressive 86.2% MO removal efficiency. Furthermore, the study explored the reusability of the rGO/Ni-BDP nanocomposite in repeated cycles of photocatalytic MO degradation under visible light irradiation. While exhibiting some decrease in efficiency over five cycles, the nanocomposite maintained a respectable degradation rate even after multiple uses. Finally, the antimicrobial properties of the nanocomposite were evaluated against both Gram-negative (*Escherichia coli*) and Gram-positive (*Staphylococcus aureus*) bacteria exhibiting a zone of inhibition measuring 23 mm and 26, respectively. The minimum inhibitory concentration (MIC) values of 2.50 µg/ml and 1.25 µg/ml for *Escherichia coli* and *Staphylococcus aureus*, respectively. The results revealed significant antibacterial activity, demonstrating the broad-spectrum efficacy of the rGO/Ni-BDP nanocomposite. This research underscores the potential of the rGO/Ni-BDP nanocomposite as a versatile material for environmental remediation and antibacterial applications.

**Supplementary Information:**

The online version contains supplementary material available at 10.1038/s41598-024-77734-7.

## Introduction

Porphyrins and analogous compounds have garnered substantial interest owing to their distinctive physical and chemical characteristics. Notably, they possess inherent photo-reactivity, rendering them valuable as natural photocatalysts. Furthermore, these molecules exhibit versatility, making them viable candidates for various applications across multiple disciplines, including electronics, physics, and materials science, due to their straightforward synthesis process, which accommodates a broad spectrum of substituent options. The impressive chemical, physical, and optoelectronic properties of porphyrins and related compounds have contributed significantly to their widespread appeal^1–10^.

Porphyrins, characterized by their extensive π-electron system, exhibit a remarkable capacity for coordination with nearly all metals within the periodic table when functioning as divalent ligands. This property enables the manipulation and optimization of the supramolecular structure and optoelectronic characteristics of these molecules through the incorporation of a metallic center within the porphyrin ring, thereby yielding a metalloporphyrin compound^11,12^.

Water contamination has emerged as a global menace to humanity owing to the rapid advancement of industrialization worldwide^13,14^. Sustainable Development Goal (SDG) 6.3 emphasizes the critical need of providing uncontaminated water and developing safe, sustainable water purification solutions from environmental, industrial, and societal viewpoints. Poor drinking water quality is estimated to account for 80% of global diseases and 50% of child fatalities. Due to the significant contamination of water resources, there has been a growing effort to develop safe and effective water treatment technologies in recent decades^15,16^.

Water contaminated by various industrial processes, including textiles, leather, pigments, rubber, and plastics, often retains residues of synthetic dyes. Notably, certain types of these synthetic dyes exhibit toxicity and persistence, posing significant environmental and public health concerns due to their presence in natural water bodies. Various technological approaches have been evaluated to determine their effectiveness in eliminating synthetic dyes from water. These methods include adsorption, photocatalytic degradation, bio-degradation, ion exchange, membrane filtration, flocculation, and ozonation^17–19^. Methyl orange (MO) is an anionic organic dye, belongs to the azo-dyes family, is commonly known as a carcinogenic and/or mutagenic substance. In addition, it is widely used in paper manufacturing, pharmaceutical, food industries, as well as in research laboratories as an acid-indicator^20^.

The chemical reduction process employed in treating graphene oxide can result in the removal of multiple functional groups, yielding graphene sheets with diminished functional group content, commonly referred to as reduced graphene oxide (rGO). Although exhibiting inferior electrical conductivity compared to pristine graphene sheets, rGO is deemed a versatile material suitable for photocatalytic applications. Additionally, rGO is better at adsorbing aromatic pollutants because it has a higher number of defects, lower oxygen content, a larger surface area, and a higher level of hydrophobicity. It is also easy for it to form stable aqueous dispersions^21–24^.

Photocatalysis is considered a pivotal strategy in the pursuit of environmentally friendly chemical processes due to its inherent advantages, including diminished ecological hazards, enhanced process security, and minimized energy requirements^25^.

The incorporation of graphene within composites has the potential to yield novel design and developmental prospects for future catalysts. Consequently, the modification of rGO could facilitate its utilization in various practical applications. It is noteworthy that the attachment of nanoparticles onto the graphene surface significantly enhances its photocatalytic capabilities, thereby facilitating the rapid and effective decomposition of pollutants upon exposure to visible light irradiation. Furthermore, the attachment of graphene to porphyrin and/or metalloporphyrins through covalent or non-covalent bonding is expected to enable efficient interfacial electron transfer and long-range transportation across the surface interface, thereby allowing for the tuning of the resultant nanocomposite’s catalytic activity^26,27^.

Many reported studies are focused on using a powerful adsorbent for photocatalytic degradation of toxic dyes either by the integration between carbonic materials (i.e. graphene oxide) and other nanoparticles (i.e. magnetite, ZnO)^28,29^ or using only nanoparticles (i.e. tricobalt tetroxide)^30^.

The nanocomposite formed from the combination of graphene oxide and porphyrin exhibits exceptional properties due to the integration of distinctive attributes from each material. By incorporating graphene’s singular characteristics onto the surface of porphyrin, the overall performance of the composite was significantly improved. It was found that using graphene as a base for porphyrin/metalloporphyrin made the electrical properties and cooperative links between porphyrin/metalloporphyrin and graphene much better. This is important because there are many examples in the literature of porphyrins and their linked metal complexes being used to change graphene oxide^27,31–33^. Accordingly, it is suggested that combining porphyrins with rGO may result in multifunctional carriers exhibiting impressive properties and diverse functionalities. In light of this notion, porphyrin-based rGO systems emerge as prime contenders, and their inherent capabilities have yet to be fully realized.

In our previous study^34^, we’ve succeeded to investigate for the first time the applicability of reduced graphene oxide/porphyrin (rGO-P) nanocomposite in the photocatalytic degradation of Congo red (CR) dye in the wastewater as well as identify the rGO-P antimicrobial effect. The fabricated rGOP nanocomposite shows substantial potential in the effective removal of pollutant dyes as well as significant antibacterial and antifungal properties.

**Fig. 1:**
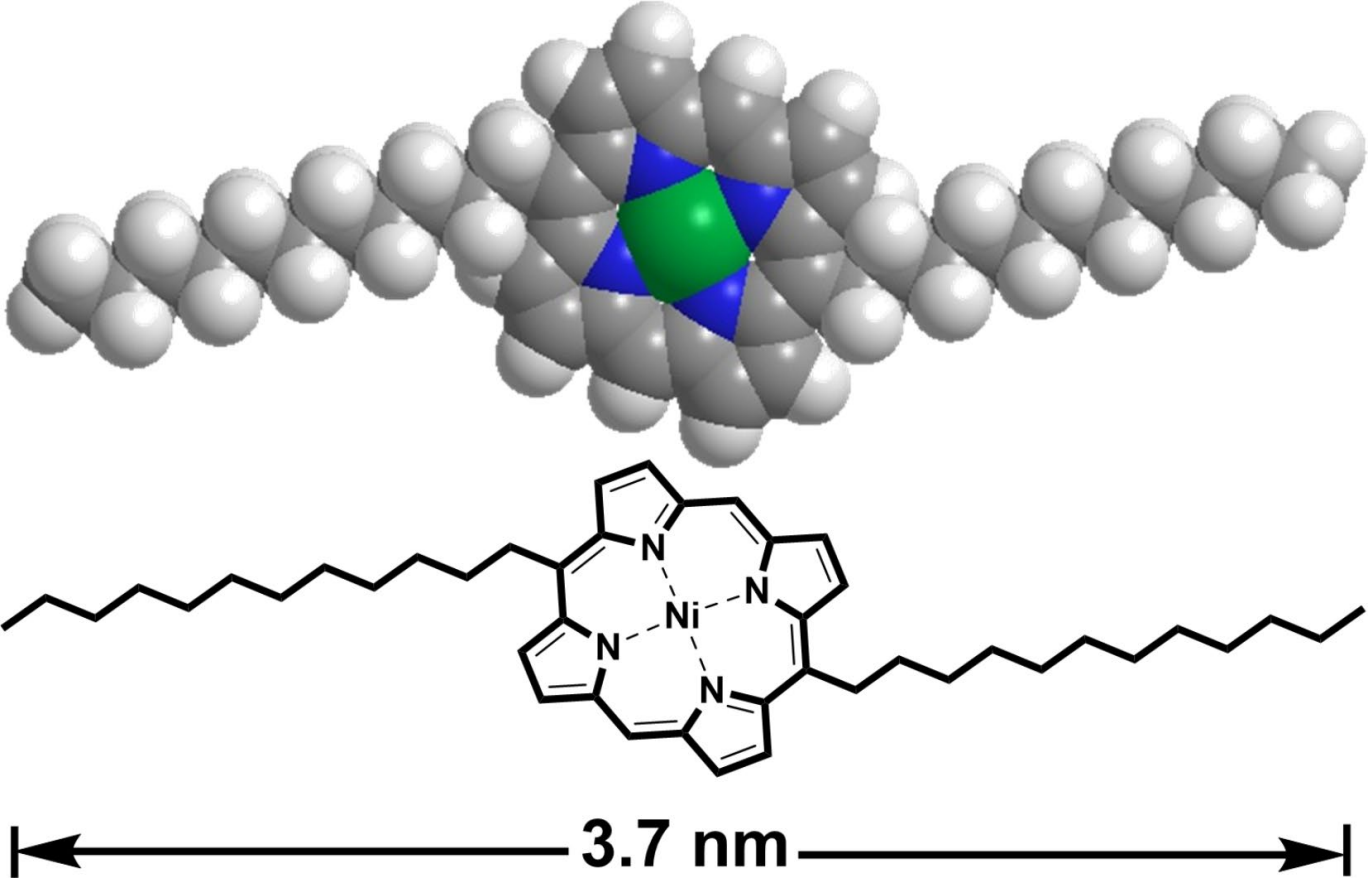
Chemical structure of Nickel-5,15-bisdodecylporphyrin (Ni-BDP). In the context of Ni-BDP modeling, the constituent elements comprising carbon-hydrogen-nitrogen (C-H-N) and Nickel (Ni) atoms are depicted using distinct colors, specifically grey-white-blue for the former and green for the latter.

This research presents the successful synthesis and comprehensive characterization of a nickel-based porphyrin compound, specifically Nickel-5,15-bisdodecylporphyrin (Ni-BDP), Fig. 1. Furthermore, it describes the fabrication and thorough examination of a rGO-loaded Ni-BDP nanocomposite. The study also explores the potential application of this nanocomposite as a photocatalytic agent for wastewater treatment, focusing on the removal of methyl orange (MO), a common anionic dye contaminant, from an aqueous environment. Additionally, the investigation examines the antimicrobial properties of the fabricated rGO/Ni-BDP nanocomposite against various bacterial strains, including both gram-positive and gram-negative species. This is work is an extension to our novel work related to utilization of porphyrin/metalloporphyrin derivatives loaded nanocarbons as a promising photocatalyst for the effective removal of pollutant dyes (water treatment) as well as significant antibacterial and antifungal properties and hence preserve our environment from dangerous contaminants.

## Experimental section

### Chemicals

The identification and sources of all the chemicals used in this study are presented in Table S1, where all the chemicals are in the reagent grade and used as purchased without any further purification. All the characterization techniques employed are thoroughly elucidated in the supplementary information section for further reference and comprehensive understanding.

The Nickel-5,15-bisdodecylporphyrin (Ni-BDP) molecule was synthesized under dark conditions. Prior to the synthesis, all solvents, namely dichloromethane, chloroform, and hexane, were meticulously dried and distilled employing molecular sieves 4 Å. The synthesis of the Ni-BDP molecule was conducted under a nitrogen atmosphere, utilizing thoroughly dried glassware preheated in an oven at 90 °C prior to use. Moreover, column chromatography was conducted utilizing silica gel (spherical, neutral, 63–200 μm, Kishida Chemicals Co., Ltd.) to purify the synthesized molecule.

### Synthesis of nickel-5,15-bisdodecylporphyrin (Ni-BDP) nanoparticles

The synthesis of Ni-BDP was accomplished through a procedure previously described in our published research^7^. Specifically, the reaction involved the metallization of 5,15-bisdodecylporphyrin (BDP) (0.3157 g, 0.488 mmol) with Nickel(II) acetylacetonate Ni(acac)_2_ (0.17 g, 0.659 mmol), using toluene as the solvent and maintaining a temperature of 120 °C for a period of 24 h. This process was carried out under dark conditions to prevent any potential degradation or contamination of the resulting compound. The efficacy of the metalation process was verified through the utilization of thin-layer chromatography (TLC). Following this verification, the reaction mixture was transferred to aqueous solution, whereupon it underwent chloroform extraction. Subsequent washing of the organic phase with water and threefold treatment with sodium chloride solution, culminating in drying via sodium sulfate. Ultimately, recrystallization from a solvent combination comprising chloroform and methanol yielded a reddish-purple powder weighing approximately 0.3 g, corresponding to an 86% yield. The detailed depiction of the synthesis procedures is presented in Scheme S1.

### Synthesis of reduced graphene oxide (r-GO) nanosheets

The chemical oxidation and exfoliation process for the graphite precursor was conducted utilizing an enhanced Hummers’ method, employing a comparable methodology to previous reports^34,35^. A solution consisting of 600 mL of concentrated sulfuric acid (H_2_SO_4_) and 75 mL of concentrated phosphoric acid (H_3_PO_4_) was prepared. Five grams of finely ground graphite powder were then added to the mixture, which was maintained at room temperature. Next, 30 g of potassium permanganate (KMnO_4_) were slowly introduced into the mixture over the course of one hour while continuously stirring. The mixture was then stirred for another 24 h at room temperature after this step was done. The resultant concentrated liquid was then broken up and put into a 5-liter jar with 4 L of cold distilled water. The beaker was put in a bath of cold water that was set to 2 °C and stirred all the time. Upon visual inspection of the sample, which exhibited a distinct yellowish color consistent with the successful synthesis of GO, we proceeded to introduce 100 mL of a 15% H_2_O_2_ solution into the diluted mixture. Subsequent processing involved decanting the resulting product and subjecting it to triple washing with 1 M HCl/H_2_O to remove impurities, followed by additional triple washing with distilled water to attain neutral pH levels.

The reduction step proceeded by transferring the purified GO into a 2 L beaker. 10 g of ascorbic acid were utilized as the reducing agent. The GO dispersion was gradually supplemented while being violently agitated at a temperature of 80 °C. The combination was allowed to undergo a reaction for a period of 24 h. Following this protocol, the ultimate black reduced graphene oxide (rGO) result was rinsed thrice with distilled water and subsequently filtered using a vacuum. To achieve additional purification, the procedure entailed the separation and cleansing of the rGO by employing a combination of water and ethanol (H_2_O/EtOH). This process was conducted to eliminate any residual by-products and contaminants. The final powdered form of rGO was then dried for a duration of 48 h (24 h under ambient temperature then 24 h at 40 °C). A visual representation of the entire synthesis procedure has been provided in Figure S1.

### Reduced graphene oxide-nickelporphyrin (rGO/Ni-BDP) nanocomposite preparation

The preparation of rGO/Ni-BDP nanocomposite was accomplished through a methodology previously described^34^ as illustrated in Figure S2. Initially, 0.33 milligrams of Ni-BDP were dissolved in chloroform (20 milliliters). Following ultrasonic treatment for 10 min, 3 mg of rGO were introduced into the solution while undergoing continuous sonication for five hours. The resulting suspension was allowed to settle for two hours before being subjected to centrifugation. Subsequently, the upper 5% of the supernatant liquid was removed through decantation, followed by filtration utilizing a membrane filter with a mesh size of 0.1 micrometers (MILLIPORE). To eliminate any unreacted porphyrin molecules, the precipitated material was washed with 100 milliliters of chloroform. Ultimately, the synthesized composite was dried under vacuum conditions and reserved for future use.

### Photocatalytic performance of rGO/Ni-BDP nanocomposite on methyl orange degradation

**Fig. 2:**
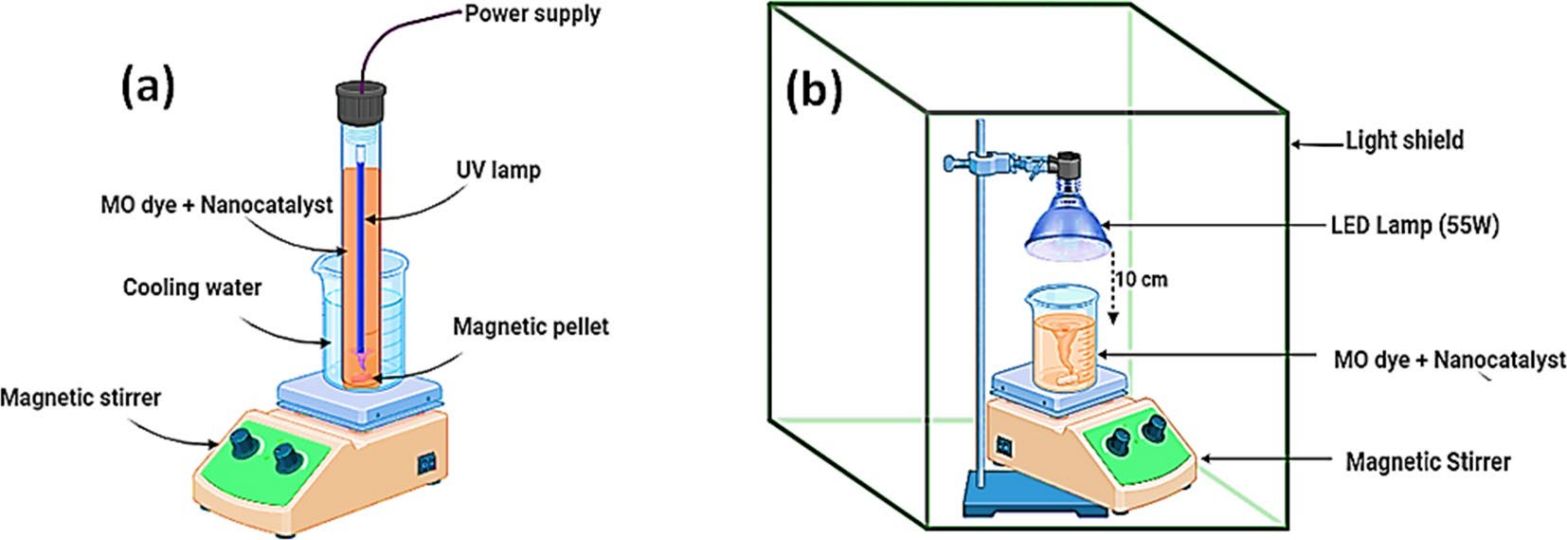
The photocatalytic setups for (**a**) UV, and (**b**) visible light, irradiation.

The photocatalytic decomposition of MO dye was successfully achieved through the utilization of a UV lamp and a novel catalyst called rGO/Ni-BDP nanocomposites. The employed UV reactor featured a cylindrical design with a glass body, measuring 3 cm in diameter and 27 cm in length, as depicted in Fig. 2. A thin layer of aluminum foil was applied to cover the reactor. Subsequently, 50 mL of contaminated solution were introduced into the photoreactor for treatment.

The UV light irradiation source utilized for this experiment was a commercially available Philips TUV 11WG11 T5 UV-C lamp, which is a high-pressure mercury vapor lamp emitting radiation at a mean wavelength of 254 nm. This lamp was submerged within the contaminated solution, while the photoreactor was maintained at a temperature of approximately 15 °C through the use of a cold-water bath. In contrast, the visible light used for irradiation was generated by a custom-built apparatus consisting of 52 white LEDs with a nominal power output of 55 watts and an emission spectrum spanning the range of 400–800 nm. To minimize losses due to irradiation, the LEDs were surrounded by aluminum reflectors. The irradiation was applied from above, with a fixed distance of 10 cm separating the light source from the photoreactor.

A 10 mg nanocomposite was introduced into a 50 mL aqueous solution containing MO (initial concentration (C_0_) of 10 mg/L). The mixture was then stirred continuously at 25 °C for 30 min. in the dark, permitting the attainment of equilibrium between adsorption and desorption processes. At pre-designated time intervals, a filtered syringe was employed to collect a 1-mL sample from the MO suspension. The rate of MO degradation was then determined by utilizing a UV-visible spectrophotometer (Agilent Technologies Cary 60 UV-visible) with a maximal absorption wavelength (λ_max_) of 464 nm to measure the changes in MO concentration during irradiation. Deionized water was employed as the control medium in this study^20^.

The MO dye and the rGO/Ni-BDP nanocomposite catalyst were initially introduced to a glass cylindrical reactor, followed by exposure to UV irradiation. A one-milliliter sample of the MO solution was subsequently withdrawn from the reactor using a syringe at a predetermined interval. The sample was then subjected to centrifugal separation for a duration of twenty minutes before being analyzed via spectrophotometry at a wavelength of 464 nm^36^.

The photodecomposition efficacy, quantified by removal percentage, was determined through application of the following mathematical formula^37^.

1 $${\rm Removal\,Percentage} = 1-\left(\frac{Ct}{C0}\right)*100$$

The initial concentration of MO in mg/L, denoted by “C_0_”, whereas “C_t_” represents the concentration at time (t). A comprehensive examination of the operational conditions for photocatalytic degradation was conducted, encompassing the starting concentrations of pollutants and pH levels.

### Antimicrobial activity of rGO/Ni-BDP nanocomposite

The antimicrobial effectiveness of the rGO/Ni-BDP nanocomposite was evaluated through the agar-disc diffusion technique^38^, which involves testing its ability to inhibit the growth of various micro-organisms. Specifically, the study examined the inhibitory effects of rGO/Ni-BDP on both Gram-negative *E. coli* (ATCC 25922) and Gram-positive *S. aureus* (ATCC 25923). To facilitate comparisons, control samples consisting of conventional antibiotic discs impregnated with gentamicin (CN, at a concentration of 10 µg per disc) and measuring 6.0 mL in diameter were also included for evaluation purposes.

The minimum inhibitory concentrations (MIC) of the most effective antimicrobial specimens were quantified through the application of the serial dilution method within a Luria-Bertani (LB) agar-based growth medium^38^.

Both positive and negative controls were used in this approach; the positive control contained both the growth medium and the pathogenic bacteria. Furthermore, the generated nanoparticles were incorporated into the evaluation procedure, initially at a concentration of 20.0 µg/mL. Following a 24-hour incubation period conducted at a controlled temperature of 36.0 ± 1.0 °C, the MIC values were determined^39^.

### Characterization techniques

HR-TEM measurements were conducted using the JEM-2100 F apparatus from JEOL, Japan. The dispersive Raman microscopy was utilized to examine the chemical composition of rGO powder samples by Raman spectroscopy. This analysis was conducted utilizing the Senterra II equipment from Bruker, Germany. The Raman spectra were collected with a spectral resolution of 4 cm^− 1^ and were then examined using spectrophotometry. A Nd: YAG laser, doped with neodymium and yttrium aluminum garnet, was used for the experiment. The laser, sourced from Bruker, Germany, produced a wavelength of 532 nm and a power output of 10 mW. The Raman excitation source was focused with a Nikon 20 objective lens. The data was collected in a time span of 1000 milliseconds, using a diaphragm illumination zone of 50 × 50 μm during the measuring process.

^1^H-NMR spectroscopy experiment was conducted utilizing a high-field NMR spectrometer operating at a frequency of 500 megahertz, manufactured by JEOL and originating from Japan. Tetramethylsilane (TMS), employed as an internal reference compound, enabled precise calibration of the NMR signal intensities. Additionally, mass spectrometry analyses were carried out employing a time-of-flight mass spectrometer, specifically the Shimadzu AXIMA-CFR MALDI-TOF instrument.

## Results and discussion

### Ni-BDP synthesis and characterization

The targeted Ni-BDP molecule depicted in Fig. 1 was synthesized according to the procedure outlined in Scheme S1. Afterward, the molecule was then characterized using various analytical methods, including^1^H-NMR, UV-Vis spectroscopy, elemental analysis and high-resolution mass spectrometry, which is completely consistent with our previous reported study^7^. The full characterization data are presented in the SI section and displayed in Supplementary Figures S3-S5.

### Synthesis and characterization of rGO nanosheets

The synthesis of rGO nanosheets was successfully achieved utilizing our previously established protocol^34^ as illustrated in Figure S1.

**Fig. 3:**
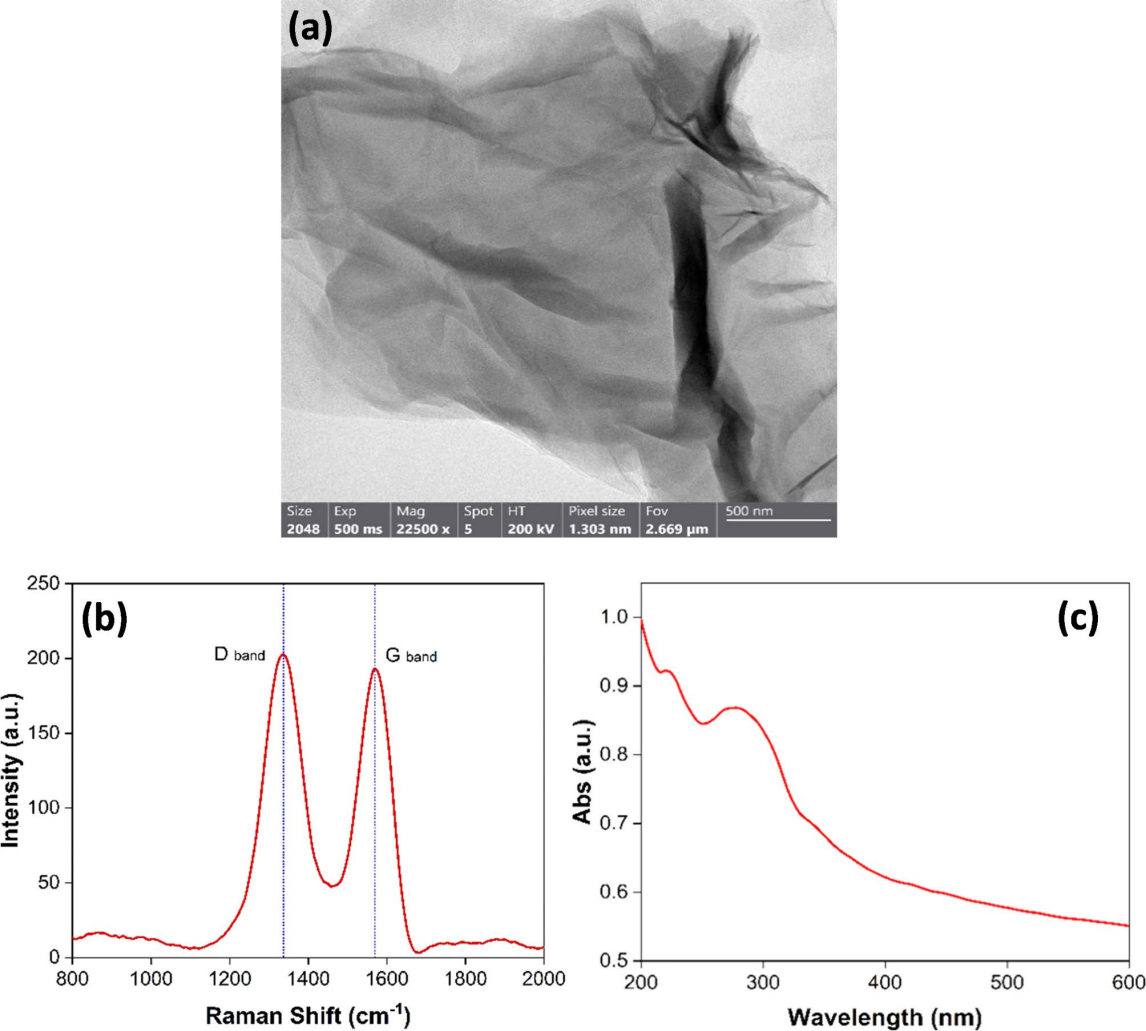
(**a**) TEM image, (**b**) Raman spectrum, (**c**) UV-Visible absorption spectrum of the produced rGO.

Figure 3a displays TEM image of rGO, showcasing a notable and effective exfoliation of graphite into individual rGO sheets. Furthermore, the surface of rGO reveals no residual reactants or by-products, signifying a pristine synthesis procedure. Figure 3b illustrates the Raman spectroscopy for the synthesized rGO, exhibiting a prominent G-band peak at 1582 cm^− 1^, congruent with the spectral fingerprint of graphite. Furthermore, a wide D-band peak appears around 1350 cm^− 1^, which is a distinctive feature of rGO with relative intensity ratio *I*_D_/*I*_G_ of 1.15. The existence of these separate peaks provides indisputable evidence of the successful production of rGO^40–44^.

As shown in Fig. 3c, rGO has a UV-Vis range. It was found that the absorption peak at 284 nm is caused by electronic changes (π → π*) that happen in the material’s aromatic carbon-carbon bonds^44,45^. The observation suggests the development of complex molecular structures exhibiting elevated levels of conjugation. Furthermore, the detection of a relatively weak absorption peak at 224 nm is attributed to the presence of residual oxygen-containing functional groups, which induce n→π* electronic transitions^46,47^.

As depicted through TEM, Raman, and UV analyses, it is evident that rGO has undergone significant structural alterations, deviating from its original graphite configuration. The reduction process yields clusters of rGO sheets that exhibit random packing arrangements. Furthermore, the Raman spectral data confirms the successful synthesis of thin layers of rGO with dimensions less than 10 nm in thickness, comprising only a few atomic layers^40,42–44^.

### Fabrication and characterization of rGO/Ni-BDP nanocomposite

The synthesis of the rGO/Ni-BDP nanocomposite was achieved via a previously established protocol^34^, as depicted in Figure S2. The process involved exposing the Ni-BDP solution to sonication in the presence of rGO. The characteristics of the resulting composite were further analyzed using UV-Vis spectroscopy to verify the interactions between the Ni-BDP and rGO components. In addition, we examined the UV-Vis spectral patterns of both rGO and Ni-BDP molecules before and after their combination to explore any alterations caused by their complexation.

**Fig. 4:**
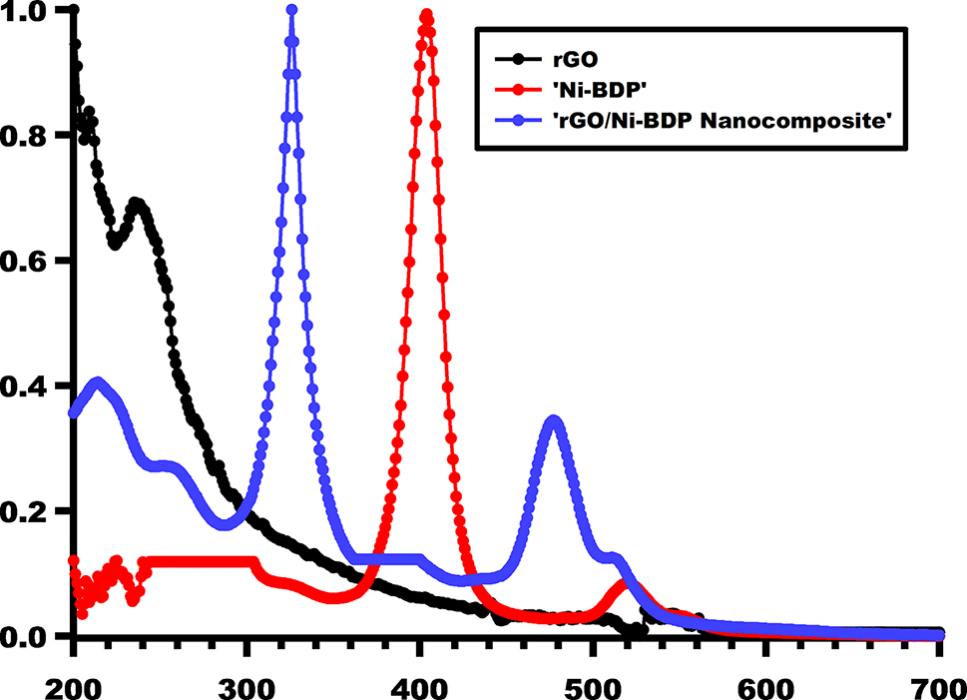
UV-Visible spectra (in CHCl_3_) of rGO, Ni-BDP, and rGO/Ni-BDP nanocomposite.

Figure 4 illustrates the UV-Visible spectral profiles of the Ni-BDP molecule, rGO, and their composite (rGO/Ni-BDP). Notably, the composite spectrum shows the peaks of both Ni-BDP and rGO.

exhibiting a pronounced blue shift relative to the Ni-BDP spectrum. Specifically, the Soret peak undergoes a blue shift from 403 to 326 nm, while the Q-bands exhibit a similar shift from 517 to 477 nm, as illustrated in Fig. 4. The phenomenon of a blue shift in observations has been found to be attributable to non-covalent interactions, specifically π-π stacking, occurring between Ni-BDP and rGO. This observation is consonant with previous research findings on this subject^24,48–51^.

Since the band gab energy (E_bg_) can be calculated using the following formula^52^:

$$E_{bg} = 1240 / \lambda (eV)$$.

where λ = is the maximum absorption wavelength in nm, observed from UV-Visible spectrum. Therefore, the band gab energy (*E*_*bg*_) for Ni-BDP and rGO/Ni-BDP nanocomposite equals 3.08 and 3.80, respectively.

**Fig. 5:**
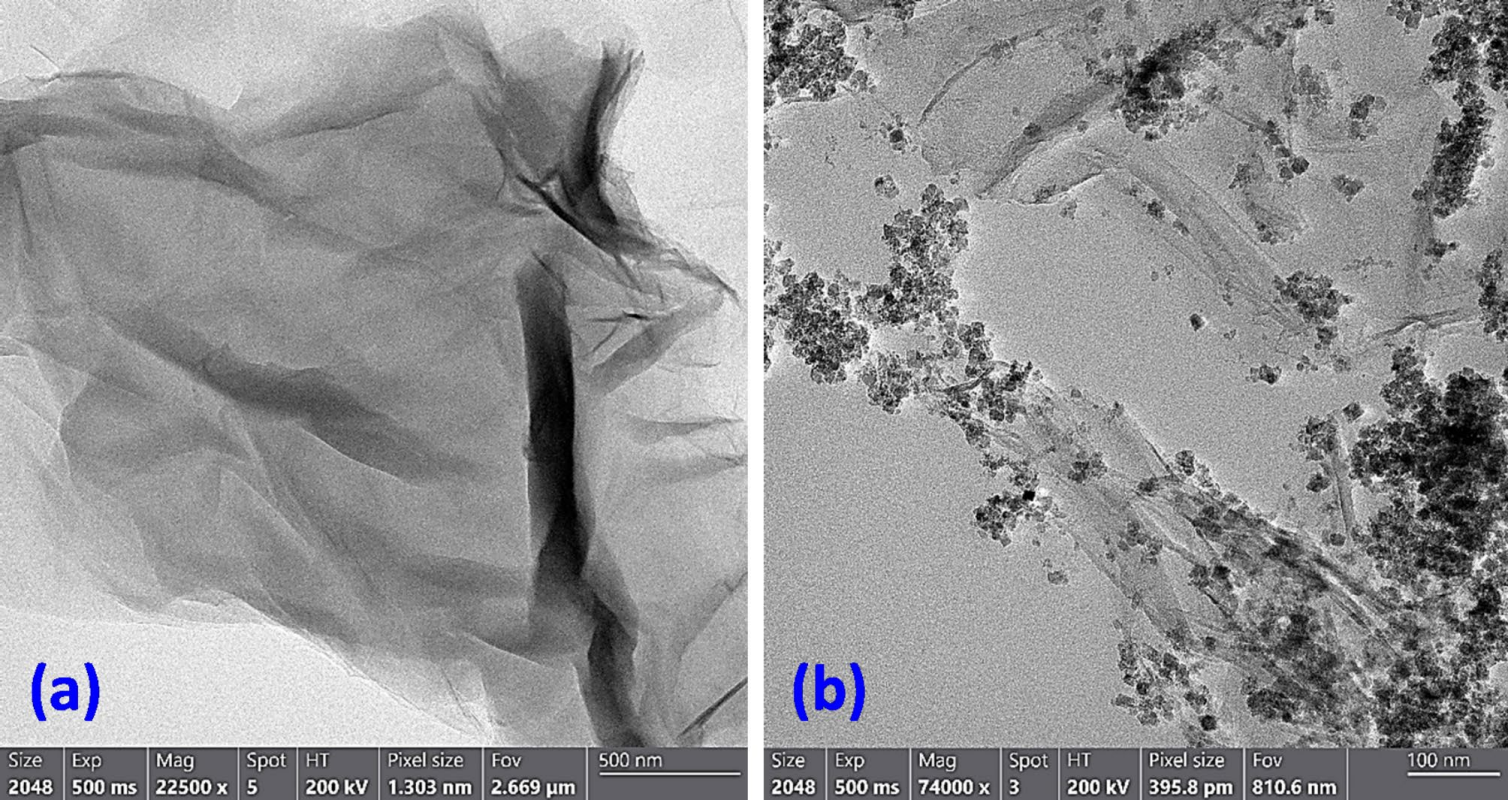
HR-TEM images of (**a**) rGO nanosheets and (**b**) rGO-NiBDP NPs.

The structural properties of the rGO/Ni-BDP nanocomposite were also investigated by HR-TEM. Figure 5a, b presents HR-TEM images depicting the morphology of rGO prior to and subsequent to its interaction with Ni-BDP, respectively. The presence of black dots in Fig. 5b provides clear evidence of the effective attachment of Ni-BDP molecules onto the rGO surface. This confirms the occurrence of π–π stacking interactions between Ni-BDP and rGO. This is in accordance with the UV-Visible measurements, and it is also congruent with our previous results^34^.

### Photocatalytic performance of rGO/Ni-BDP nanocomposite against MO degradation under different conditions

**Fig. 6:**
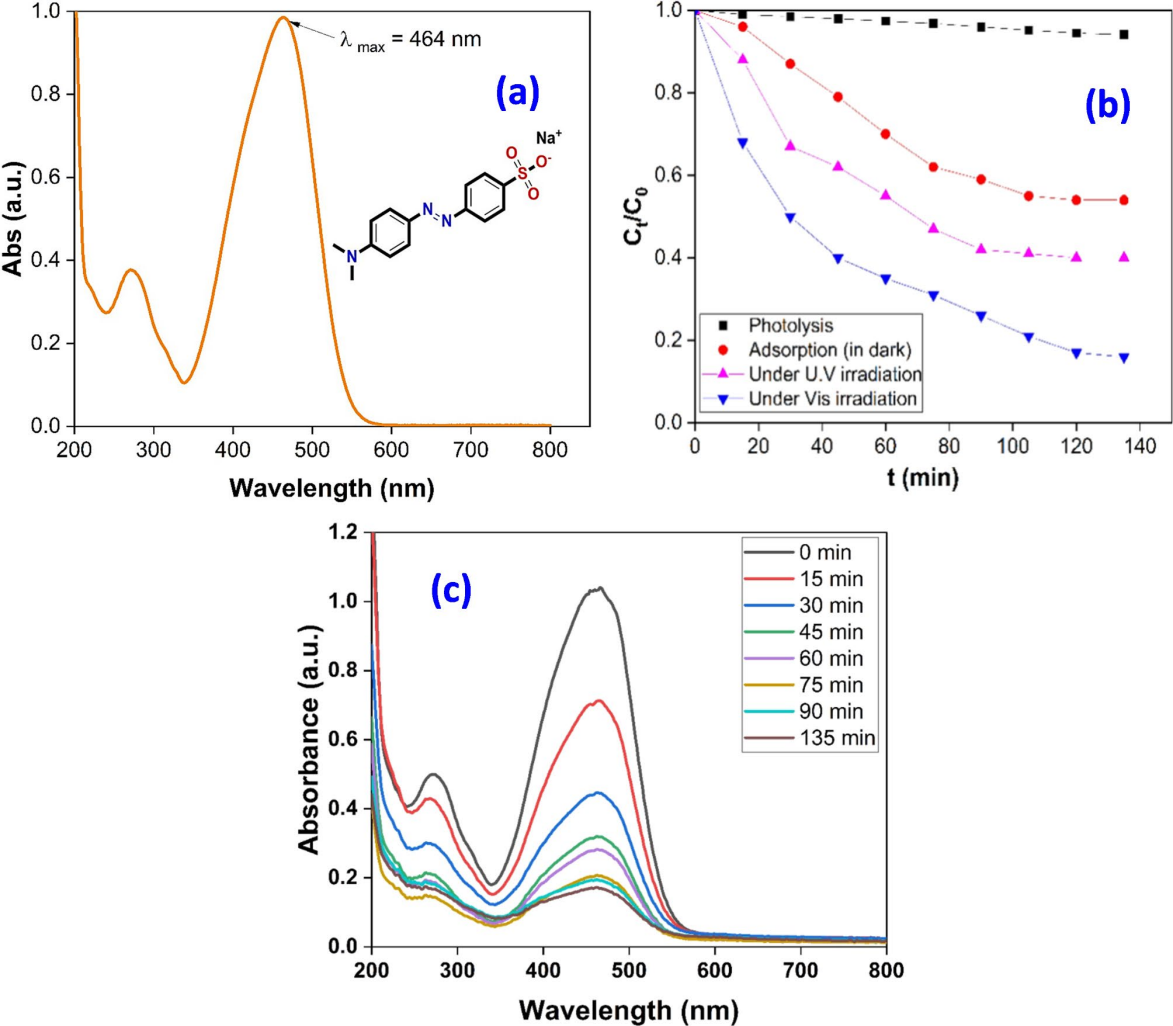
(**a**) UV-Visible spectrum of MO (10 mg/L), (**b**) Removal % of MO via: photolysis without catalyst, adsorption in dark on rGO/Ni-BDP nanocomposite surface, photocatalysis under UV irradiation, and photocatalysis under visible light irradiation. (**c**) UV-Visible spectrum illustrating the complete degradation of MO.

The elimination of MO was closely monitored at its optimal absorbance wavelength of λ_max_ = 464 nm^53^. A graph illustrating the UV-visible spectrum of MO at a concentration of 10 mg/L is presented in Fig. 6a. It has been observed that the adsorption-based removal process, conducted in the absence of illumination, resulted in a removal efficiency of approximately 43% over a period of 135 min when using the rGO/Ni-BDP nanocomposite. In contrast, the photodegradation of MO under ultraviolet radiation facilitated by the rGO/Ni-BDP nanocatalyst achieved a removal rate of 59% within the same timeframe, as depicted in Fig. 6b. Notably, the photocatalytic activity of the rGO/Ni-BDP nanocatalyst under visible light was found to reach an impressive 86.2%, as shown in Fig. 6b. The exceptional photocatalytic properties of the rGO/Ni-BDP nanocomposite can be attributed to the presence of a metal-semiconductor junction within the composite structure, which enables efficient charge separation and light absorption. Figure 6c demonstrates the UV-Visible spectrum for the complete degradation of MO.

#### Effect of different pH values on dye removal

**Fig. 7:**
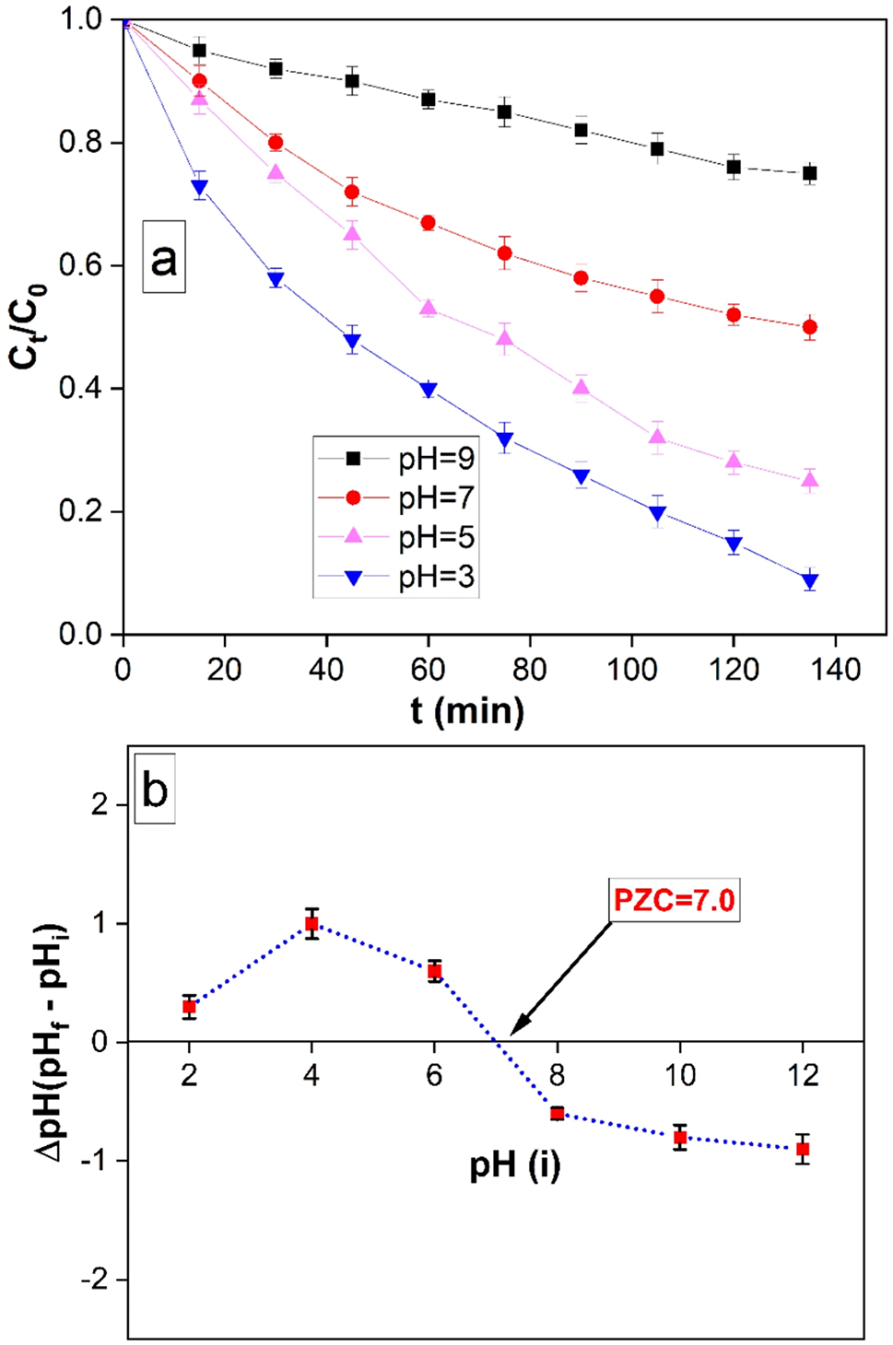
(**a**) MO removal rates changed over time at four different solution pHs (3.0, 5.0, 7.0, and 9.0) (10 mg of rGO/Ni-BDP nanocomposite in 50 ml of 10 mg/L MO at 25 °C), (**b**) Point of zero charge of rGO/Ni-BDP nanocomposite at different pH values.

The influence of solution pH on the nanocomposite’s removal efficacy was examined. A controlled experimental setup was employed to examine the effects of varying initial pH values (pH 3.0, 5.0, 7.0, and 9.0) on the removal process. Specifically, the experiments were conducted using a fixed amount of the nanocomposite (10 mg), a predetermined concentration of the model organic compound (MO; 10 mg/L), and a constant temperature of 25 °C. The removal process was monitored for a duration of 90 min. Visual representations of the data enabled the assessment of the changes in MO removal percentage over time at each tested pH level. Notably, the highest degree of MO removal at equilibrium was achieved at a pH value of 3.0, as depicted in Fig. 7a.

The point of zero charge (PZC) of the rGO/Ni-BDP nanocomposite was investigated by introducing 0.01 g of the composite into 50 milliliters of a 0.01 molar sodium chloride solution. The solutions were intentionally adjusted to achieve specific pH values of 3, 5, 7, and 9. Following this, the samples underwent stirring at a rate of 200 rpm for a period of 48 h, after which pH measurements were taken after the magnetic separation of the rGO/Ni-BDP nanocomposite particles.

The PZC was determined through the construction of a graph depicting the relationship between the final and initial pH values (Fig. 7b). The PZC point was identified as the specific pH value at which there exists minimal deviation between the final and initial pH readings. When the pH of the solution reaches the point of zero charge (PZC), the surface charge of the photocatalyst becomes neutral, minimizing electrostatic interactions between the photocatalyst surface and ions like MO ions. The rGO/Ni-BDP NPs exhibit a positive charge on their surface when the pH is below the point of zero charge (PZC) and a negative surface charge when the pH exceeds the PZC. The determined PZC value for the rGO/Ni-BDP NPs is 6.7. The findings clarify the optimal conditions for the degradation of MO through photocatalysis, which happens at a pH of 3.0 (as shown in Fig. 7a). At this pH, the rGO/Ni-BDP NPs carry a positive charge on their surface, allowing for favorable interactions between the positively charged catalyst and the negatively charged MO. As a result, the photocatalytic effectiveness is significantly improved.

Another point that among all the first transition elements, we’ve chosen nickel, as Ni has the highest electronegativity (Pauling electronegativity scale^54^), which increases the electropositivity of BDP molecule as well as the rGO/Ni-BDP NPs, therefore, enhancing the degradation of MO dye. This agrees with the results found at pH = 3 (Fig. 7a).

#### Effect of dye concentration and amount of nanocatalyst on degradation efficiency

**Fig. 8:**
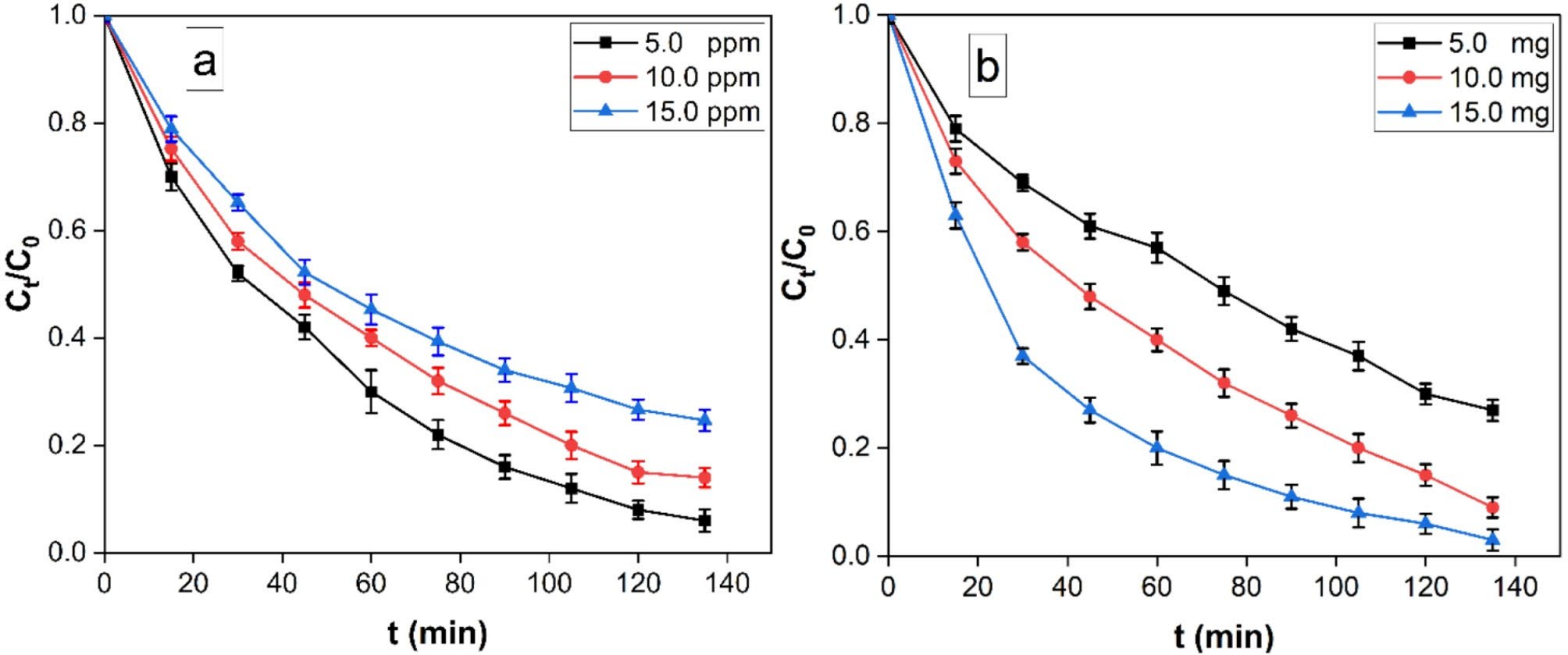
(**a**) The variation of removal % as a function of contact time at different initial MO concentration (5–15 mg/L) with 10.0 mg rGO/Ni-BDP nanocomposite at pH 3.0, (**b**) Effect of the nanocatalyst amount (5–15 mg) on removal efficacy (10 mg/L MO solution, Temperature = 25 °C and pH 3.0).

The preliminary concentration of MO has been found to significantly impact its removal efficacy. A graphical representation of the variation in removal percentages over time at distinct initial MO concentrations (5.0, 10.0, and 15.0 ppm) is depicted in Fig. 8a. The findings demonstrate an inverse correlation between degradation efficiency and MO concentration.

Furthermore, the effect of varying rGO/Ni-BDP nanocomposite dosages on MO removal efficacy under visible light illumination was examined by employing diverse photocatalyst amounts ranging from 5 to 20 mg against a constant MO concentration of 10 mg/L (Fig. 8b). The results indicate a corresponding increase in removal efficiency with increasing photocatalyst dosage from 5 to 20 mg. Notably, it is observed that the maximum removal efficiency of MO reaches 95% when utilizing 20 milligrams of rGO/Ni-BDP nanocomposite. Govindhan, G., et al. (2023), succeeded to synthesize rGO/CuCoO_2_ materials with enhanced photocatalytic degradation of 94% against Methylene blue (MB) dye using 20 mg of rGO/CuCoO_2_ catalyst, in which case composites were more efficient with environment-friendly when compared to CuCoO_2_ nanoparticles^55^. On the other hand, Govindhan, G., et al. (2024) conclude that (rGO/ZnNiO_2_ (75/25) nanocomposite degradation efficiency is 88% at 80 min^56^. The enhancement in removal efficiency with increased catalyst quantity may be attributed to the augmentation of available active surface area or active sites of the photocatalyst relative to the volume of the MO solution^57^.

#### Kinetic studies

The MO removal rate was computed using the following equation:

2 $$- ln{\text{ }}C_{t} /C_{0} = - {\text{ }}kt$$

**Fig. 9:**
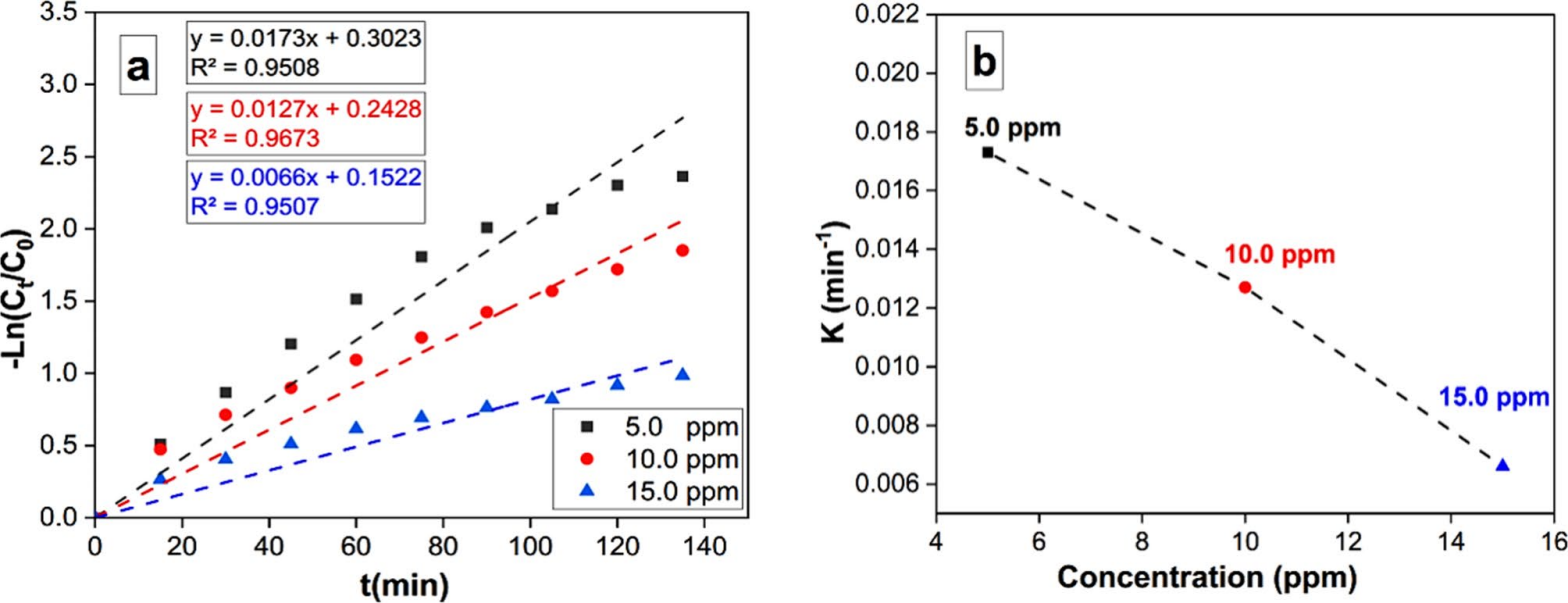
(**a**) Pseudo-first-order reaction model for MO degradation under visible-light irradiation (MO initial concentrations (5–15 mg/L), (**b**) The relation of apparent pseudo-first-order rate constants vs. initial concentration of MO.

In this context, C_t_ and C_0_ represent the residual and initial concentrations of MO, respectively. The duration of removal is denoted by *t*, while *k* signifies the removal rate constant. A graphical representation of the correlation between -ln (C_t_/C_0_) and *t* is depicted in Fig. 9a. The results indicate that the removal reaction kinetics conform to pseudo-first-order rates. Notably, an enhancement in the initial MO concentration leads to an escalation in the apparent pseudo first order rate constants, as illustrated in Fig. 9b. This dependence of rate constants on MO concentration is in accordance with previously documented research findings^58,59^.

#### Mechanism of MO photocatalysis

As mentioned in several studies of works of literature^60–63^, the possible mechanism is as follows; When photons, in the form of light rays, interact with a material and have energy that is equal to or greater than its bandgap, electrons in the conduction band (CB) move to the valence band (VB) by crossing the bandgap, resulting in the creation of positive holes, Consequently, this results in the production of reactive oxygen species (ROS), which is the most important consequence of photocatalysis because it has an effect on the environment and is therefore utilized in the degradation of MO dye. It is believed that visible light will cause electron-hole pairs to form on the photocatalyst’s surface, which will then lead to photocatalytic degradation^64–66^.

The chemical reactions underlying the photocatalytic degradation of MO can be succinctly encapsulated within the parameters outlined below (Eqs. 3–6).

3 $${\text{rGO}}/{\text{Ni}} - {\text{BDP NPs}} + hv~~~ \to ~~~{\text{rGO}}/{\text{Ni}} - {\text{BDP NPs}}\left( {{\text{e}}^{ - } _{{{\text{CB}}}} + {\text{ h}}^{ + } _{{{\text{VB}}}} } \right)$$

4 $${\text{h}}^{ + } _{{{\text{VB}}}} + {\text{rGO}}/{\text{Ni}} - {\text{BDP NPs}}~ \to ~~{\text{rGO}}/{\text{Ni}} - {\text{BDPNPs}}^{ + } \left( {{\text{Oxidation of the compound}}} \right)$$

$${\rm Or}$$

5 $${\text{h}}^{ + } _{{{\text{VB}}}} + {\text{ OH}}^{ - } \to ~{\text{OH}}^{.}$$

6 $${\text{OH}}^{.} + {\text{ MO dye }} \to {\text{ Degradation products}}$$

**Fig. 10:**
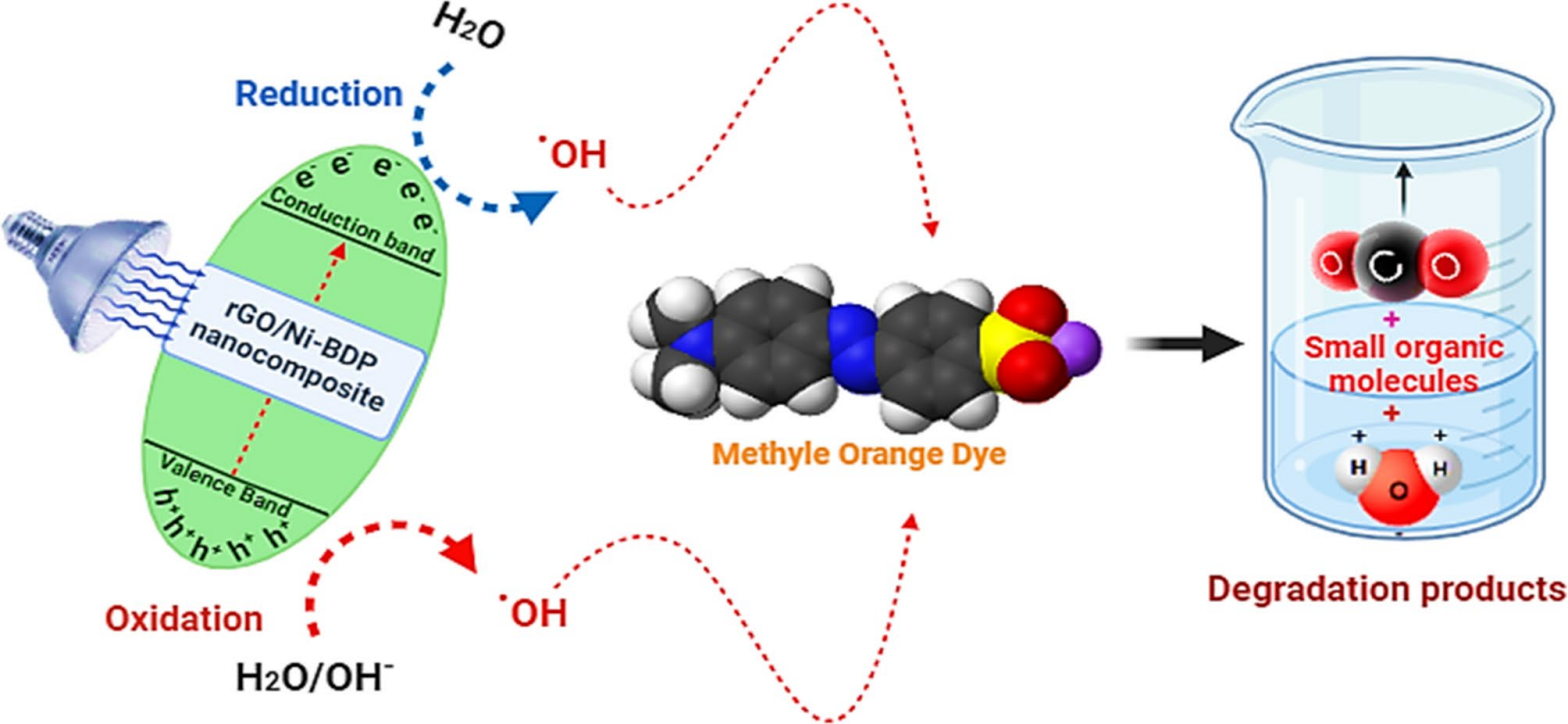
A proposed photocatalytic reaction mechanism for MO photodegradation by rGO/Ni-BDP nanocomposite.

Figure 10 elucidates the proposed mechanism with the interplay between the synthesized rGO/Ni-BDP nanoparticles and MO. Upon exposure to visible light, the rGO/Ni-BDP nanoparticles undergo excitation, yielding charge carriers that trigger redox reactions. Subsequently, the generated free radicals, comprising hydroxyl radical (OH^·^) and superoxide radical (O_2_^·−^), these active radicals act as powerful oxidizing agents, efficiently breaking down MO molecules to produce the ultimate oxidation products, resulting in the formation of smaller organic compounds. ROS are generated by trapping photogenerated electrons or holes in the solution via several species, especially dissolved oxygen, hydroxyl ions, and water molecules. The essential ROS species include hydroxyl (^·^OH), superoxide (^·^O_2_), and singlet oxygen^1^O_2_).

**Fig. 11:**
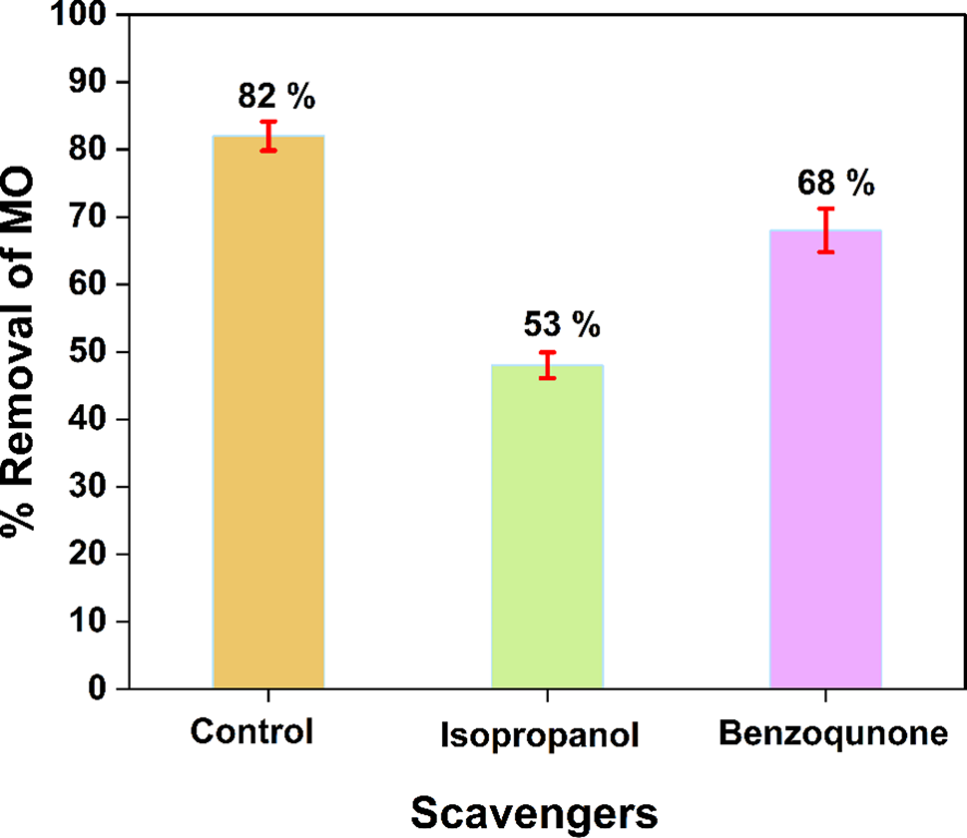
Effect of scavengers on MO degradation over rGO/Ni-BDP nanocomposite.

The effect of scavengers on the photocatalytic degradation process was studied to identify the most active species. The isopropanol and benzoquinone were employed to trap OH^·^ and ^−^O_2_^·^, respectively^67^. Figure 11 depicts the efficiency of photodegradation of MO by rGO/Ni-BDP in the absence and presence of scavengers at 5 ppm concentration. In the presence of benzoquinone and isopropanol, the efficiency reduced from 82% to roughly 68% and 53%, respectively. When isopropanol was added, the photodegradation of CR dye by the rGO/Ni-BDP was decreased (53%), suggesting that the OH^•^ radical was the primary species responsible for MO degradation. Furthermore, adding benzoquinone lowered the degradation rate by 68%, revealing that the ^−^O_2_^·^ radical played a prominent role in MO degradation^68^.

#### Reusability of the rGO/Ni-BDP nanocomposite

The reusability of the nanocatalyst is considered essential for practical application, as it greatly enhances the economic viability and feasibility of the approach^69^. This suggests that suitable reusability of rGO/Ni-BDP nanocomposite in successive cycles of application is an important aspect to study. Therefore, a supplementary investigation was undertaken to examine the potential reusability of rGO/Ni-BDP nanocomposite in the context of photocatalytic reduction of MO dye when subjected to Visible light irradiation. The photocatalytic reuse experiments were conducted using identical settings as those stated in the earlier evaluation of photocatalytic activity. The dye degradation rates in the initial and subsequent 5 cycles were found to be 82.0%, 80.4%, 78.3%, 71.6%, 63.2%, and 49.8%, respectively as shown in Figure S6. Before its utilization in the subsequent cycle, rGO/Ni-BDP nanocomposite were subjected to centrifugation, followed by washing with deionized water and thereafter allowed to undergo overnight drying. The decrease in residual activity seen over six cycles may be attributed to the agglomeration of nanomaterials^70^.

### Antimicrobial activity of synthesized rGO/Ni-BDP nanocatalyst

The in-vitro zone of inhibition (ZOI) test as illustrated in Figure S7, showed that rGO/Ni-BDP NPs at a concentration of 20 µg/ml were more effective against *Staphylococcus aureus*, exhibiting a zone of inhibition measuring 26 mm and minimum inhibitory concentration (MIC) values of 1.25 µg/ml. Similarly, this formulation also displayed potent antimicrobial properties against *Escherichia coli*, yielding a ZOI of 23 mm and MIC values of 2.50 µg/ml, as presented in Table 1.

**Table 1:** In-vitro zone of inhibition ZOI (mm) and minimal inhibitory concentration MIC (µg/ml) of rGO/Ni-BDP NPs, against gram-positive and gram-negative bacteria.

Bacterial strains	ZOI (mm) of rGO/Ni-BDP NPs (10.0 µg/ml)	ZOI (mm) of rGO/Ni-BDP NPs (20.0 µg/ml)	CN ZOI (mm)	MIC (µg/ml) of rGO/Ni-BDP NPs
*S. aureus*	12.0 ± 0.426	26.0 ± 0.712	15.0 ± 0.322	1.25
*E. coli*	10.0 ± 0.532	23.0 ± 0.415	21.0 ± 0.425	2.50

The experimental outcomes indicate that the fabricated rGO/Ni-BDP NPs exhibited enhanced antimicrobial activity against Gram-positive bacteria relative to their effect on Gram-negative microorganisms. This disparity in efficacy may arise from distinct structural and compositional characteristics inherent to the cell walls of Gram-negative versus Gram-positive bacteria^71^.

**Fig. 12:**
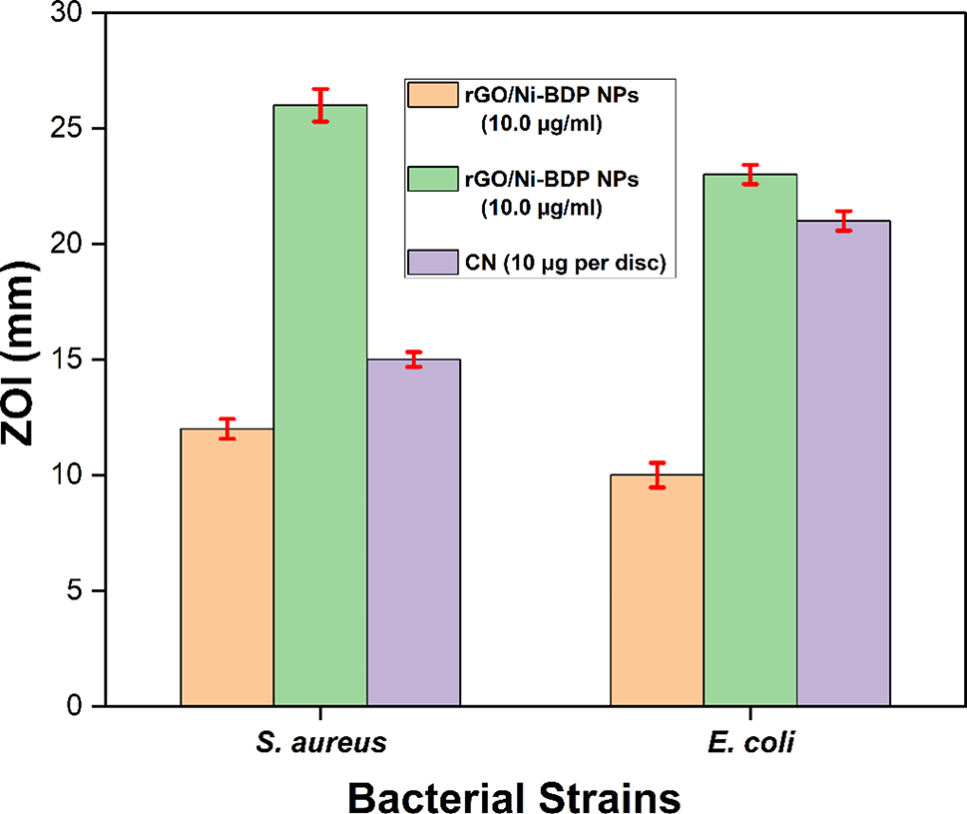
The bar graph shows zone of inhibition produced by rGO/Ni-BDP NPs (10 and 20.0 µg/ml) against standard gentamycin antibiotic (10.0 µg/disc).

Gram-positive bacteria exhibit a thick layer of peptidoglycan, to which teichuronic and teichoic acids are chemically bonded. In contrast, Gram-negative bacteria possess a sparse covering of peptidoglycan, overlaid by an outermost layer composed of negatively charged lipopolysaccharides^71^. As a result of these factors, the synthesized rGO/Ni-BDP NPs exhibited a substantial inhibitory impact on Gram-positive bacterial species compared to Gram-negative bacterial species, as depicted in Table 1 and represented in Fig. 12.

**Fig. 13:**
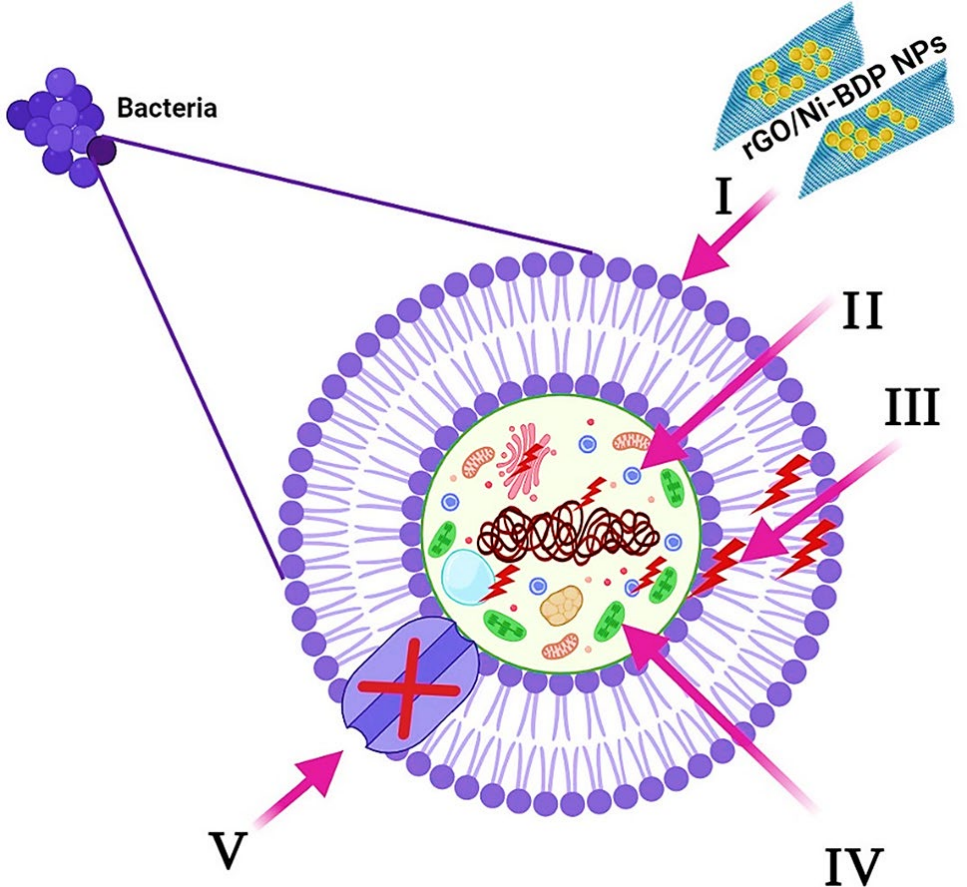
Schematic representation of the main pathways underlying the antibacterial potential of the rGO/Ni-BDP NPs: (I) Adheres of rGO/Ni-BDP NPs to and wrap the bacterial cell surface, resulting in damage of bacterial cell. (II) rGO/Ni-BDP NPs penetrate the microbial cells and affect the respective cellular machinery. (III) rGO/Ni-BDP NPs creates and increases ROS, leading to cell damage. (IV) rGO/Ni-BDP NPs modulates the cellular signal system and causing cell death. (V) Finally, rGO/Ni-BDP NPs block the ion transport from and to the microbial cells.

Figure 13 is an illustration of the possible antibacterial mechanism^20,27,72^. First, the rGO/Ni-BDP NPs start their process by attaching and wrapping themselves around the outside of the bacteria cells. This breaks down the membranes and changes the transport potential. Then, the porphyrin’s location inside the microbial cell separates all the structures inside, such as DNA, plasmids, and other important parts. After that, reactive stress caused by ROS production leads to cell death. Finally, rGO/Ni-BDP NPs stop the movement of ions to and from microbe cells.

## Conclusion

This research investigation focuses on the preparation and incorporation of the reduced graphene oxide (rGO) with nickel-5,15-bisdodecylporphyrin (Ni-BDP) nanoparticles to create a novel rGO-loaded Ni-BDP (rGO/Ni-BDP) nanocomposite. The resulting material exhibits exceptional capabilities for removing methyl orange (MO), a hazardous and carcinogenic synthetic dye, from aqueous solutions. The efficacy of the resultant rGO/Ni-BDP nanocomposite as both a catalyst and adsorbent were assessed through various parameters, including pH, initial concentration of the target dye, and quantity of the nanocomposite used. Notably, 0.01 g of the rGO/Ni-BDP nanocomposite achieved an impressive 86.2% removal efficiency of MO at a pH of 3.0. At this pH, the rGO/Ni-BDP NPs carry a positive charge on their surface, allowing for favorable interactions between the positively charged catalyst and the negatively charged MO. As a result, the photocatalytic effectiveness is significantly improved. It is also observed that the maximum removal efficiency of MO reaches 95% when utilizing 20 milligrams of rGO/Ni-BDP nanocomposite. The potential reusability of rGO/Ni-BDP nanocomposite was examined in the context of photocatalytic reduction of MO dye when subjected to Visible light irradiation. The MO dye degradation rates in the initial and subsequent 5 cycles were found to be 82.0%, 80.4%, 78.3%, 71.6%, 63.2%, and 49.8%, respectively. Furthermore, the antimicrobial properties of the nanocomposite were evaluated by determining the minimum inhibitory concentration (MIC) and zone of inhibition (ZOI) against several bacterial strains, including Gram-negative *Escherichia Coli* and Gram-positive *Staphylococcus Aureus*. The results indicate that the rGO/Ni-BDP nanocomposite exhibited remarkable antimicrobial activities against these pathogens, with ZOI values of 23 mm and 26 mm, respectively. Overall, this study highlights the promising applications of the rGO/Ni-BDP nanocomposite in the efficient removal of pollutants and its significant antibacterial properties.

## Electronic Supplementary Material

Below is the link to the electronic supplementary material.

Supplementary Material 1

## Data Availability

All data generated or analysed during this study are included in this manuscript [and its supplementary information files].
