# Publisher Correction: Development of novel reduced graphene oxide/metalloporphyrin nanocomposite with photocatalytic and antimicrobial activity for potential wastewater treatment and medical applications

**DOI:** 10.1038/s41598-025-90580-5

**Published:** 2025-02-19

**Authors:** Ahmed M. El-Khawaga, Hesham Tantawy, Mohamed A. Elsayed, Ahmed I. A. Abd El-Mageed

**Affiliations:** 1 Department of Basic Medical Sciences, Faculty of Medicine, Galala University, Galala City, 43511 Suez Egypt; 2 Head of Chemical Engineering Department, Military Technical College (MTC), Egyptian Armed Forces, Cairo, Egypt; 3 Chemical Engineering Department, Military Technical College (MTC), Egyptian Armed Forces, Cairo, Egypt; 4 Chemistry Department, Faculty of Science, Galala University, Galala City, 43511 Suez Egypt; 5 Colloids and Advanced Materials Group, Chemistry Department, Faculty of Science, Minia University, Minia, 61519 Egypt

Correction to: *Scientific Reports* 10.1038/s41598-024-77734-7, published online 13 November 2024

The original version of this Article contained errors in the Figure legends of Figure 1 and Figure 2. The legends of these Figures were inadvertently switched.

The legend of Figure 1:

“The photocatalytic setups for (**a**) UV, and (**b**) visible light, irradiation.”

now reads:

“Chemical structure of Nickel-5,15-bisdodecylporphyrin (Ni-BDP). In the context of Ni-BDP modeling, the constituent elements comprising carbon-hydrogen-nitrogen (C-H-N) and Nickel (Ni) atoms are depicted using distinct colors, specifically grey-white-blue for the former and green for the latter.”

The legend of Figure 2:

“Chemical structure of Nickel-5,15-bisdodecylporphyrin (Ni-BDP). In the context of Ni-BDP modeling, the constituent elements comprising carbon-hydrogen-nitrogen (C-H-N) and Nickel (Ni) atoms are depicted using distinct colors, specifically grey-white-blue for the former and green for the latter.”

now reads:

“The photocatalytic setups for (**a**) UV, and (**b**) visible light, irradiation.”

The original Article has been corrected.

