# Supplementary material for: Development of novel reduced graphene oxide/metalloporphyrin nanocomposite with photocatalytic and antimicrobial activity for potential wastewater treatment and medical applications
Source: Sci Rep. 2024 Nov 13;14:27916. doi: 10.1038/s41598-024-77734-7 (PMC11561097; doi:10.1038/s41598-024-77734-7)
Supplement: Supplementary file 1 — Supplementary Material 1 [file 41598_2024_77734_MOESM1_ESM.docx]

**Supplementary Information**

**Development of novel reduced graphene oxide/metalloporphyrin nanocomposite with photocatalytic and antimicrobial activity for potential wastewater treatment and medical applications**

**Ahmed M. El-Khawaga ^a,*^, Hesham Tantawy ^b^, Mohamed A. Elsayed ^c^,**

**and Ahmed I. A. Abd El-Mageed ^d,e,*^**

*^a^Department of Basic Medical Sciences, Faculty of Medicine, Galala University, Galala City 43511, Suez, Egypt.*

*^b^Head of Chemical Engineering Department, Military Technical College (MTC), Egyptian Armed Forces, Cairo, Egypt.*

*^c^ Chemical Engineering Department, Military Technical College (MTC), Egyptian Armed Forces, Cairo, Egypt.*

*^d^Chemistry Department, Faculty of Science, Galala University, Galala City 43511, Suez, Egypt.*

*^e^Colloids & Advanced Materials Group, Chemistry Department, Faculty of Science, Minia University, Minia 61519, Egypt.*

**Corresponding Authors’ E-mails:*

*ahmed.elkhawaga@gu.edu.eg and* [*ahmed.abdelmageed@gu.edu.eg*](mailto:ahmed.abdelmageed@gu.edu.eg)

***Keywords:*** *Reduced graphene oxide, Metalloporphyrins, Antimicrobial activity, Photocatalysis, Nanocomposite, Water treatment. Nickel, Methyl orange.*

**Supplementary Table(s)**

**Table S1.** Chemicals used in the experimental work.

| Chemicals | Purity | Source |
| --- | --- | --- |
| Graphite | 99.5% | NICE / India |
| Potassium Permanganate | 98% | Alpha Chemicals / India |
| Sulfuric Acid | 98% | Alpha Chemicals / India |
| Phosphoric Acid | 85% | Alpha Chemicals / India |
| Hydrochloric Acid | 36% | Alpha Chemicals / India |
| Hydrogen Peroxide | 35% | Alpha Chemicals / India |
| Ascorbic Acid | 98% | Alpha Chemicals / India |
| Ethanol | 99% | Alpha Chemicals / India |
| Methyl Orange dye | >98.0 | S.D. fine-chem. pvt. (Limited/ India) |
| Toluene | 99% | Merck |
| Nickel(II) acetylacetonate | ≥95% | Sigma Aldrich |
| Chloroform | 99.9 % | Thermo Fisher Scientific |
| Dichloromethane | ≥99.8% | Thermo Fisher Scientific |
| Tridecanal | 90% | Sigma Aldrich |
| Trifluoroacetic acid | 99.5 | Sigma Aldrich |
| Triethylamine | ≥99.5% | Sigma Aldrich |
| Hexane | ≥95% | Alpha Chemicals / India |
| Methanol | ≥99.8% | Alpha Chemicals / India |

**Supplementary Scheme(s)**

**Scheme S1.** Procedures for synthesizing Nickel-5,15-bisdodecylporphyrin (Ni-BDP) molecule.

**Supplementary Figure(s)**

**Figure S1.** Synthesis of reduced graphene oxide (rGO) ^1^.

**Figure S2.** Preparation of rGO/Ni-BDP nanocomposite.

**Ni-DBP Full characterization**

The full characterization data of Ni-BDP molecule, which is completely consistent with our previous reported study ^2^. **M.p.** 153 °C. **^1^H NMR** (500 MHz, CDCl_3_, 25 ° C, TMS): δ 9.80 (s, 2H, *meso*-H), 9.60 (d, J = 4.8 Hz, 4H, β-pyrrole-H), 9.34 (d, J = 4.8 Hz, 4H, β-pyrrole-H), 4.95 (m, 4H, CH_2_), 2.48 (m, 4H, CH_2_), 1.80 (m, 4H, CH_2_), 1.63 (m, 16H, CH_2_), 1.11 (t, 6H, CH_3_). **UV/Vis (CH_2_Cl_2_):** λ_max_ = 403, 517, 550 nm. **MS (MALDI-TOF):** *m/z* for C_44_H_60_N_4_Ni, [M^+^] calcd, 702.41; found, 702.04. **Elemental analysis:** calcd for C_44_H_60_N_4_Ni: C, 75.10; H, 8.59; N, 7.96; Ni, 8.34; found C, 74.93; H, 8.61; N, 7.82. **HRMS-ESI (m/z):** calcd for C_44_H_60_N_4_Ni, [M^+^] 702.4171, found 702.4163.

**Figure S3.** ^1^H-NMR Spectrum of Nickel-5,15-bisdodecylporphyrin (Ni-BDP) molecule.

**Figure S4.** Mass Spectrum of Nickel-5,15-bisdodecylporphyrin (Ni-BDP) molecule.

**Figure S5.** High resolution Mass Spectrum of Nickel-5,15-bisdodecylporphyrin (Ni-BDP) molecule.

**Figure S6.** Recyclability of rGO/Ni-BDP for MO degradation under visible light irradiation.


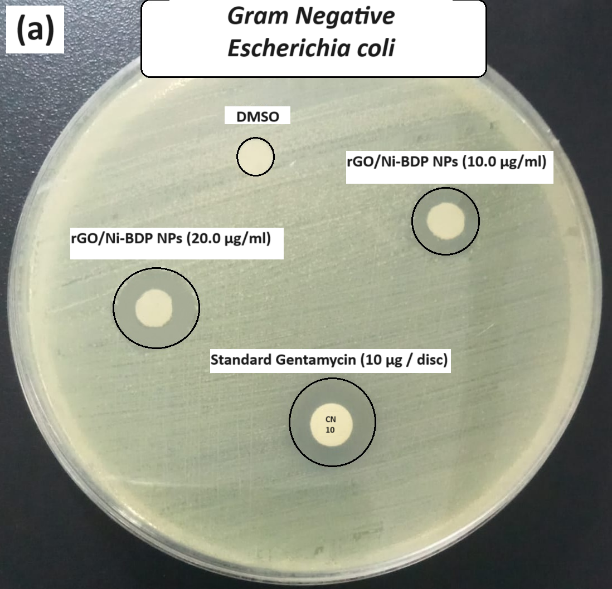

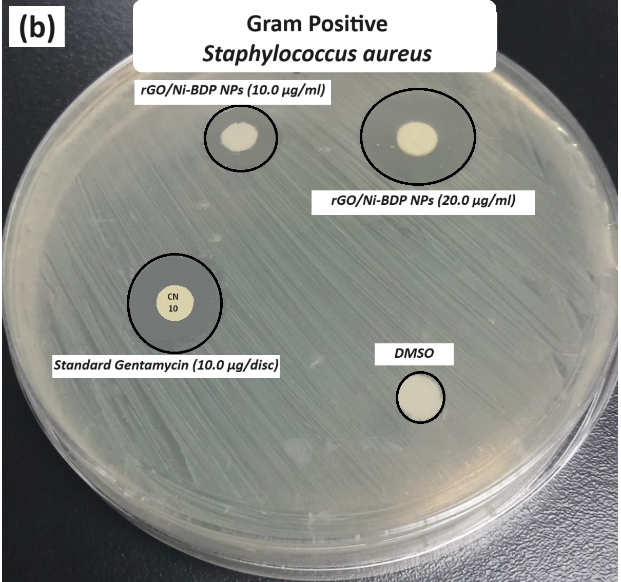


**Figure S7.** Antimicrobial activity as ZOI for rGO/Ni-BDP nanocomposite against (**a**) Gram-negative (*E. coli*), and (**b**) Gram-positive (*S. aureus*) bacteria, using standard antibiotic Gentamycin (CN) as positive control and DMSO as negative control.

**References**

1 El-Khawaga, A. M., Tantawy, H., Elsayed, M. A. & Abd El-Mageed, A. I. Synthesis and applicability of reduced graphene oxide/porphyrin nanocomposite as photocatalyst for waste water treatment and medical applications. *Sci. Rep.* **12**, 17075 (2022).

2 Abd El‐Mageed, A. I. & Ogawa, T. Single‐walled carbon nanotube absolute‐handedness chirality assignment confirmation using metalized porphyrin's supramolecular structures via STM imaging technique. *Chirality* **32**, 345-352 (2020).
